# Association Between Cerebral Blood Flow Velocity and Neurological Outcomes After Cardiac Arrest: A Retrospective Transcranial Color Flow Imaging Study

**DOI:** 10.7759/cureus.95863

**Published:** 2025-10-31

**Authors:** Saki Yamamoto, Ginga Suzuki, Tsuneyoshi Yakuwa, Saria Nishioka, Toshimitsu Kobori, Yuka Masuyama, Hibiki Serizawa, Yoshimi Nakamichi, Masayuki Watanabe, Mitsuru Honda, Yosuke Sasaki

**Affiliations:** 1 Critical Care Center, Toho University Omori Medical Center, Tokyo, JPN; 2 Department of Clinical Functional Physiology, Toho University Omori Medical Center, Tokyo, JPN; 3 Department of General Medicine and Emergency Care, Toho University School of Medicine, Tokyo, JPN

**Keywords:** clinical neurology, critical care, critical care medicine, intensive care, intensive care unit

## Abstract

Introduction: Neurological outcomes after cardiac arrest (CA) remain a challenge in critical care. Time-averaged maximum flow velocity (TAMX) measured via transcranial ultrasound has been suggested as a potential predictor of neurological prognosis, but its relationship remains unclear. This study investigated the association between TAMX, recorded using transcranial color flow imaging (TCCFI), and neurological outcomes.

Methods: This single-center retrospective study included patients admitted to the ICU with initial ventricular fibrillation (VF) following CA. Patients aged <18 years and those who developed cerebral hemorrhage or infarction during their course were excluded. The primary outcome assessed was the cerebral performance category (CPC) at discharge, with CPC 1-2 categorized as 'good CPC.' Logistic regression models were used to analyze the relationship between TAMX and good CPC, adjusting for body temperature, mean arterial pressure, pH, hemoglobin level at examination, and time from CA to examination. The TAMX was categorized into three groups (low, medium, and high), with medium as the reference for subsequent analyses.

Results: Among 41 patients, TAMX was significantly associated with good CPC in logistic regression analyses. The medium TAMX group showed significantly better neurological outcomes (CPC 1-2, 78.9%) compared with the high TAMX group (30.8%, p = 0.01). In adjusted analyses, the medium TAMX group was significantly associated with favorable CPC outcomes compared with the high group.

Conclusion: The TAMX measured via TCCFI may be associated with neurological outcomes in post-CA patients. Medium TAMX levels were associated with better neurological recovery than high levels. The TAMX may serve as a simple bedside prognostic marker, but further studies are warranted to validate these findings and clarify the underlying mechanisms.

## Introduction

Neurological outcomes after cardiac arrest (CA) remain a significant challenge in the field of critical care medicine [1,2]. Despite advancements in resuscitation techniques and post-CA management, many patients experience poor neurological recovery [1-4], which negatively impacts their quality of life and increases healthcare costs [5,6]. Identifying early and reliable predictors of neurological outcomes is crucial for guiding therapeutic interventions and improving prognosis.

Transcranial color flow imaging (TCCFI) has emerged as a promising tool for noninvasive assessment of cerebral blood velocity [7-23]. Unlike traditional transcranial Doppler (TCD), TCCFI allows angle correction and visualization of vascular anatomy, enabling more accurate measurements of blood flow velocity. While prior studies have explored the relationship between cerebral blood flow and outcomes after CA using TCD [19-23], the findings have been inconsistent, and comprehensive analyses accounting for potential confounding factors are lacking.

In particular, the role of time-averaged maximum flow velocity (TAMX) as a middle cerebral artery (MCA) blood flow parameter in predicting neurological outcomes is not well established [19-23]. Recent physiological studies have suggested that abnormalities in cerebral autoregulation may contribute to poor outcomes, with both hypoperfusion and hyperperfusion associated with neuronal damage [19-23]. However, the relationship between TAMX and neurological prognosis has not been thoroughly investigated.

This study aimed to evaluate the association between TAMX, measured using TCCFI, and neurological outcomes as assessed by the cerebral performance categories (CPC) [24] in patients resuscitated from CA. By employing a multivariate analysis framework, we sought to account for confounding factors and elucidate the role of TAMX as a potential intermediate variable linking background clinical factors to neurological outcomes. We hypothesized that medium TAMX values would be associated with better neurological outcomes than low or high values.

## Materials and methods

Design and setting

This retrospective study was performed at Toho University Omori Medical Center, a tertiary emergency medical facility affiliated with a university in Tokyo, Japan. The study protocol was approved by the Institutional Ethics Committee of Toho University Omori Medical Center (approval number: M24132). The need for written informed consent was waived owing to the retrospective design of the study. Information about the study was made available on the institutional website, and potential participants were allowed to decline participation. 

Inclusion and exclusion criteria

Patients admitted to the ICU with an initial waveform ventricular fibrillation (VF) following CA who had recorded TCCFI data between August 2018 and December 2021, were included in the study. Inclusion was restricted to patients with VF as the initial rhythm, as it is strongly associated with CA. Other initial rhythms, such as asystole or pulseless electrical activity, often include noncardiac causes of arrest, leading to variability in the extent and nature of the initial brain injury. By focusing on VF, we aimed to study a relatively homogeneous group in which the cerebral impact of CA could be more consistently attributed to a cardiac cause. The TCCFI was performed within 72 hours post-CA to assess cerebral blood flow at an early phase of post-resuscitation care. Patients aged <18 years and those who developed intracerebral hemorrhage or cerebral infarction during their course were excluded.

TCCFI measurement

**Figure 1:**
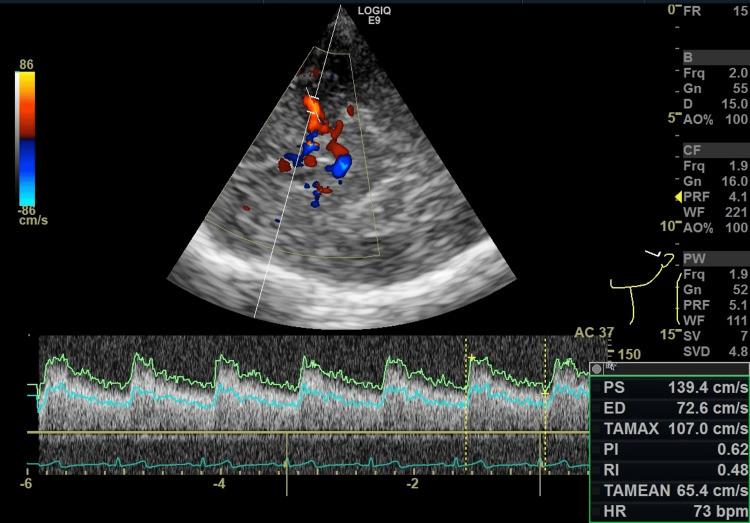
Representative TCCFI measurement of the MCA Color Doppler imaging identifies the MCA through the temporal bone window, and pulsed-wave Doppler is used to record the flow velocity waveform. The angle of insonation (AC = 37° in this example) is adjusted along the vessel course, and the TAMX is automatically calculated over one cardiac cycle by the system software (TAMX = 107 cm/s). TCCFI: Transcranial color flow imaging, TAMX: Time-averaged maximum flow velocity, MCA: Middle cerebral artery, AC: Angle correction

Cerebral blood flow was assessed using TCCFI with either a LOGIQ E9 ultrasound system (GE Healthcare, Chicago, IL, USA) equipped with an M5S-D sector probe or an Aplio i800 system (Canon Medical Systems, Otawara, JPN) equipped with a PST-28BT sector probe. The MCA was insonated through the temporal bone window, and the insonation angle was adjusted to align with the vessel course. When angle correction was necessary, it was limited to ≤ 60°, in accordance with standard Doppler principles. In most cases, the correction angle was within 30°. The TAMX was obtained from a single representative cardiac cycle showing reproducible Doppler waveforms, as illustrated in Figure 1. Measurements were performed by a single experienced sonographer.

Data extraction

Clinical data were extracted from electronic medical records and institutional databases. Variables included demographic information (age, sex), clinical characteristics (witnessed status, bystander CPR, arrest time, cause of arrest, and time from CA to examination), treatment details (use of extracorporeal membrane oxygenation (ECMO)), vital signs (body temperature (BT) and mean arterial pressure (MAP) at examination), and laboratory data (pH at admission and examination, hemoglobin (Hb) level at examination, and TAMX).

Outcome

The primary outcome was the cerebral performance category (CPC) score at discharge. The CPC scores of 1-2 were defined as 'good CPC,' whereas scores of 3-5 were categorized as 'poor CPC' [24]. The CPC score was determined by the attending physician.

Statistical analysis

Continuous variables were presented as medians and interquartile ranges (IQRs) and compared using the Kruskal-Wallis test. Categorical variables were presented as percentages and compared using the chi-square test or Fisher’s exact test. The TAMX was considered an intermediate variable between background factors and outcomes. A stepwise modeling approach was used to evaluate the influence of background factors on TAMX, the association between TAMX and good CPC, and the impact of confounding factors on TAMX.

Model 1 included background factors as independent variables with good CPC as the dependent variable. In model 2, TAMX was added as an independent variable to assess its association with good CPC. Finally, body temperature, mean arterial pressure, pH, Hb level at examination, and time from CA to examination were identified as confounders for TAMX [7,19-23]. These variables were included in model 3, which evaluated good CPC as the dependent variable, with background factors, TAMX levels, and confounding factors as independent variables.

The TAMX cutoff values were determined using X-Tile software version 3.6.1 (Yale School of Medicine, New Haven, CT, USA) [25,26], categorizing patients into three groups: low, medium, and high. The medium group was designated as the reference category in multivariate regression analyses. All analyses were performed using R version 4.2.0 (R Foundation for Statistical Computing, Vienna, AUT), and statistical significance was set at p < 0.05. Because of the retrospective nature and limited number of eligible cases, no formal sample size calculation or power analysis was performed.

## Results

A total of 43 patients were initially included, and after excluding two patients who developed cerebral hemorrhage or infarction, 41 were analyzed. The baseline characteristics of the study population are summarized in Table 1. The median age was 60 years (IQR 52-67), and most patients were male (92.8%). Among all cases, 68.3% of CA were witnessed, 63.4% received bystander CPR, and 58.5% underwent ECMO. The most common cause of CA was acute coronary syndrome (ACS) (53.7%), followed by cardiomyopathy (34.2%).

**Table 1:** Baseline characteristics Values are expressed as median (IQR) or n (%). Differences between groups were tested using the Kruskal–Wallis test for continuous variables (test statistics shown as H with degrees of freedom) and Fisher’s exact test for categorical variables (test statistics not applicable). IQR: Interquartile range; ACS: Acute coronary syndrome; ECMO: Extracorporeal membrane oxygenation; BT: Body temperature; MAP: Mean arterial pressure; Hb: Hemoglobin; TAMX: Time-averaged maximum flow velocity

Parameters	All (n = 41)	Low group (n = 9)	Medium group (n = 19)	High group (n = 13)	Test statistic	p-value
Age, years	60.0 (52.0-67.3)	65.0 (62.8-72.5)	55.0 (48.8-61.5)	56.0 (51.3-72.8)	7.9	0.02
Male, n (%)	38 (92.8)	8 (88.9)	18 (94.7)	12 (92.3)	–	0.86
Witness, n (%)	28 (68.3)	4 (44.4)	15 (79.0)	9 (69.2)	–	0.19
Bystander CPR, n (%)	26 (63.4)	3 (33.3)	16 (84.2)	7 (53.9)	–	0.02
Arrest time, min	37.0 (20.0-45.0)	40.0 (37.0-50.3)	32.0 (18.5-45.0)	35.0 (19.5-42.5)	4.0	0.14
pH at arrival	7.1 (7.0-7.2)	7.1 (7.0-7.2)	7.2 (7.0-7.3)	7.2 (7.1-7.2)	1.2	0.56
Cause of arrest					–	0.51
ACS, n (%)	22 (53.7)	3 (33.3)	10 (52.6)	9 (69.2)		
Cardiomyopathy, n (%)	14 (34.2)	5 (55.6)	6 (31.6)	3 (23.1)		
Hyper/hypokalemia, n (%)	3 (7.3)	0 (0)	2 (10.5)	1 (7.7)		
Hypothermia, n (%)	2 (4.9)	1 (11.1)	1 (5.3)	0 (0)		
ECMO, n (%)	24 (58.5)	8 (88.9)	7 (36.8)	9 (69.2)	–	0.02
BT at examination, ℃	34.4 (34.1–35.7)	34.4 (34.0–35.0)	34.6 (34.3–35.8)	34.3 (34.1–35.9)	1.4	0.49
MAP at examination, mmHg	80.0 (73.3–90.0)	76.7 (70.0–78.3)	83.3 (76.7–90.0)	80.0 (70.0–91.7)	3.4	0.18
pH at examination	7.4 (7.4–7.4)	7.4 (7.4–7.4)	7.4 (7.4–7.4)	7.4 (7.3–7.4)	1.5	0.48
Hb at examination, g/dL	11.3 (10.0–13.3)	11.3 (10.1–13.3)	11.5 (10.3–14.0)	10.1 (9.0–11.8)	5.3	0.07
Arrest to examination time, h	27.0 (21.5–48.3)	27.0 (19.3–43.3)	28.0 (22.5–48.0)	26.0 (23.0–49.5)	0.7	0.72
TAMX, cm/s	49.0 (31.0–69.5)	20.9 (20.1–23.6)	47.5 (45.7–56.0)	95.1 (71.0–142.2)	34.3	< 0.01

At the time of the TCCFI examination (median 27 h after arrest), the median TAMX was 49.0 cm/s (IQR 31.0-69.5). Other vital signs and laboratory values are shown in Table 2.

**Table 2:** Outcomes Values are expressed as median (IQR) or n (%). Differences between groups were tested using the Kruskal–Wallis test for continuous variables (test statistics shown as H with degrees of freedom) and Fisher’s exact test for categorical variables (test statistics not applicable). CPC: Cerebral performance category; IQR: Interquartile range

Parameters	All (n = 41)	Low group (n = 9)	Medium group (n = 19)	High group (n = 13)	Test statistic	p-value
CPC	3.0 (1.0-5.0)	5.0 (1.0-5.0)	1.0 (1.0-3.0)	5.0 (2.5-5.0)	6.3	0.04
CPC 1 or 2, n (%)	22 (53.7)	3 (33.3)	15 (78.9)	4 (30.8)	–	0.01

In model 1, which included background factors only (Table 3), bystander CPR was significantly associated with good CPC (p = 0.03), while other factors were not. In model 2, which added TAMX as an independent variable, TAMX was significantly associated with good CPC (odds ratio = 0.996, 95% CI 0.992-0.999, p = 0.01). In model 3, which further adjusted for potential confounders (BT, MAP, pH, Hb, and time from CA to examination; Table 4), the TAMX remained independently associated with higher odds of good CPC (odds ratio = 0.994, 95% CI 0.990-0.997, p < 0.01).

**Table 3:** Logistic regression analyses for good CPC featuring background factors and background factors with TAMX CPC: Cerebral performance category; TAMX: Time-averaged maximum flow velocity; ECMO: Extracorporeal membrane oxygenation

Parameters		Odds ratio	95% CI	p-value
Background factors (model 1)	Age	1.005	0.993-1.018	0.42
	Male	0.918	0.527-1.599	0.76
	Witness	0.885	0.645-1.213	0.45
	Bystander CPR	1.507	1.070-2.122	0.03
	Arrest time	0.995	0.982-1.008	0.46
	pH at arrival	1.474	0.450-4.829	0.53
	Cause of arrest	0.876	0.754-1.017	0.09
	ECMO	0.898	0.642-1.256	0.53
Background factors and TAMX (model 2)	Age	1.001	0.989-1.013	0.92
	Male	0.783	0.466-1.317	0.36
	Witness	0.938	0.700-1.255	0.67
	Bystander CPR	1.349	0.977-1.863	0.08
	Arrest time	0.990	0.978-1.003	0.13
	pH at arrival	1.284	0.432-3.811	0.66
	Cause of arrest	0.868	0.757-0.996	0.05
	ECMO	0.915	0.673-1.243	0.57
	TAMX	0.996	0.992-0.999	0.01

**Table 4:** Logistic regression analyses for good CPC featuring background factors, background factors with TAMX, and confounding factors CPC: Cerebral performance category; TAMX: Time-averaged maximum flow velocity; ECMO: Extracorporeal membrane oxygenation; BT: Body temperature; MAP: Mean arterial pressure; Hb: Hemoglobin

Parameters (background factors, TAMX, and confounding factors for model 3)	Odds ratio	95% CI	p-value
Age	0.994	0.981-1.008	0.41
Male	0.840	0.496-1.421	0.52
Witness	0.919	0.674-1.253	0.60
Bystander CPR	1.370	1.000-1.878	0.06
Arrest time	0.987	0.973-1.000	0.07
pH at arrival	0.986	0.303-3.210	0.98
Cause of arrest	0.873	0.758-1.006	0.07
ECMO	0.967	0.650-1.438	0.87
BT at examination	1.096	0.887-1.354	0.41
MAP at examination	1.009	0.995-1.023	0.21
pH at examination	0.175	0.007-4.534	0.30
Hb at examination	0.969	0.891-1.055	0.48
Arrest to examination time	1.005	0.994-1.017	0.37
TAMX	0.994	0.990-0.997	< 0.01

The TAMX was categorized into three groups using X-Tile, namely low (<30.8 cm/s), medium (30.8-65.3 cm/s), and high (>65.3 cm/s). Group comparisons revealed that the medium TAMX group had the highest proportion of bystander CPR (p = 0.02) and the highest rate of good CPC (78.9%), compared with 33.3% in the low group and 30.8% in the high group (p = 0.01). In multivariate regression analysis (Table 5), the medium TAMX group was independently associated with higher odds of good CPC compared with the high group (odds ratio = 0.483, 95% CI 0.363-0.643, p < 0.01). No significant difference was observed between the low and medium groups (odds ratio = 0.685, 95% CI 0.469-1.000, p = 0.06). In summary, moderate TAMX values were associated with better neurological outcomes than both low and high TAMX levels.

**Table 5:** Logistic regression analyses for good CPC featuring background factors, background factors with TAMX group, and confounding factors CPC: Cerebral performance category; TAMX: Time-averaged maximum flow velocity; ECMO: Extracorporeal membrane oxygenation; BT: Body temperature; MAP: Mean arterial pressure; Hb: Hemoglobin

Parameters	Odds ratio	95% CI	p-value
Age	1.001	0.989-1.013	0.88
Male	0.785	0.491-1.256	0.32
Witness	0.864	0.658-1.133	0.30
Bystander CPR	1.267	0.951-1.687	0.12
Arrest time	0.989	0.978-1.001	0.09
pH at arrival	1.642	0.576-4.683	0.36
Cause of arrest	0.886	0.778-1.009	0.08
ECMO	1.159	0.811-1.657	0.43
BT at examination	1.029	0.843-1.256	0.78
MAP at examination	1.008	0.996-1.020	0.19
pH at examination	0.048	0.002-0.932	0.06
Hb at examination	0.974	0.900-1.053	0.51
Arrest to examination time	1.005	0.995-1.015	0.32
TAMX low	0.685	0.469-1.000	0.06
TAMX medium	(reference)	(reference)	
TAMX high	0.483	0.363-0.643	< 0.01

## Discussion

Early evaluation of cerebral blood flow velocity using TCCFI was performed in patients with cardiac-origin CA. Multivariate analysis revealed that TAMX was independently associated with good CPC, with medium cerebral blood flow velocity showing significantly better neurological outcomes than high levels.

Comparison with previous research

Several studies have examined the relationship between cerebral blood flow parameters measured by TCD and outcomes after CA [19-23]. However, findings have been inconsistent. Wessels et al. reported higher MCA flow velocities in survivors than in non-survivors at both four and 72 hours after resuscitation [23], whereas Lemiale et al. and Hoedemaekers et al. found no significant differences [21,22]. These discrepancies likely reflect differences in study populations and severity; the shorter arrest duration in Wessels et al. suggests milder cases, while the inclusion of noncardiac etiologies in some studies may have introduced heterogeneity. Moreover, several prior investigations relied on small sample sizes and univariate analyses, limiting their conclusions [21-23]. Other studies, such as those by Doepp et al., Heimburger et al., and Bisschops et al., explored associations between flow velocities and CPC but focused on different arterial sites or failed to adjust for confounding factors [19,20,27].

Strengths and clinical implications

Unlike prior TCD-based research, the present study is the first to examine the association between MCA blood flow velocity and neurological outcomes using TCCFI. Compared with TCD, TCCFI enables angle correction and visualization of vascular anatomy, providing more accurate velocity measurements [7]. This study also advances the field by analyzing TAMX, a parameter not previously studied in this context, and identifying a potential U-shaped association between cerebral blood flow and outcomes. Patients with excessively high TAMX levels showed poorer neurological recovery, possibly reflecting hyperperfusion injury from impaired autoregulation. Although differences in the low-TAMX group did not reach significance, the trend suggests that both hypo- and hyperperfusion may be detrimental. These findings underscore the clinical importance of maintaining optimal cerebral blood flow and highlight TCCFI’s potential as a bedside tool to guide individualized post-resuscitation management.

Interpretation

The observed relationship between TAMX and neurological outcomes likely reflects disturbances in cerebral autoregulation [28]. High TAMX levels may indicate hyperperfusion due to loss of autoregulatory control, which can exacerbate neuronal injury. This suggests that maintaining cerebral blood flow within a physiological range could promote neurological recovery and may serve as a therapeutic target in post-CA care.

Limitations

This study has several limitations. First, it is a small, single-center, retrospective study, which limits generalizability. Second, TCCFI requires technical expertise, and all measurements were performed by a single experienced operator, preventing assessment of interobserver variability. Third, although TAMX was significantly associated with neurological outcomes, it may yield false-positive results, similar to other diagnostic tests; therefore, multimodal evaluation, including electroencephalography, should be considered [29]. Finally, we did not evaluate longitudinal changes in TAMX or validate outcomes across multiple time points, which could provide further insight into dynamic cerebral hemodynamics after resuscitation.

## Conclusions

For patients with cardiac arrest, TAMX levels measured in the MCA using TCCFI may be associated with favorable neurological outcomes. Further research is required to determine whether interventions aimed at maintaining TAMX levels within the medium range can improve neurological outcomes. The TCCFI may serve as a valuable noninvasive adjunct to existing neuroprognostication methods, providing real-time insight into cerebral hemodynamics in post-CA care.
